# Prevalence and Characterization of Carbapenem-Hydrolyzing Class D β-Lactamase-Producing *Acinetobacter* Isolates From Ghana

**DOI:** 10.3389/fmicb.2020.587398

**Published:** 2020-11-13

**Authors:** Alafate Ayibieke, Ayumi Kobayashi, Masato Suzuki, Wakana Sato, Samiratu Mahazu, Isaac Prah, Miyuki Mizoguchi, Kyoji Moriya, Takaya Hayashi, Toshihiko Suzuki, Shiroh Iwanaga, Anthony Ablordey, Ryoichi Saito

**Affiliations:** ^1^Department of Molecular Microbiology, Tokyo Medical and Dental University, Tokyo, Japan; ^2^Antimicrobial Resistance Research Center, National Institute of Infectious Diseases, Tokyo, Japan; ^3^Department of Environmental Parasitology, Tokyo Medical and Dental University, Tokyo, Japan; ^4^Department of Molecular Virology, Tokyo Medical and Dental University, Tokyo, Japan; ^5^Department of Infection Control and Prevention, The University of Tokyo Hospital, Tokyo, Japan; ^6^Department of Bacterial Pathogenesis, Tokyo Medical and Dental University, Tokyo, Japan; ^7^Department of Bacteriology, Noguchi Memorial Institute for Medical Research, University of Ghana, Accra, Ghana

**Keywords:** carbapenem-hydrolyzing class D β-lactamases, carbapenem-resistant *Acinetobacter*, multilocus sequence typing, whole-genome sequencing, *Acinetobacter baumannii*, OXA-type beta-lactamase

## Abstract

Multidrug resistance, especially carbapenem resistance in *Acinetobacter* bacteria is a global healthcare concern. However, available data on the phenotypic and genotypic characteristics of *Acinetobacter* isolates from West Africa, including Ghana is scanty. Our aim was to investigate the antibiotic resistance profile and genotypic characteristics of *Acinetobacter* isolates from Ghana and to characterize carbapenemase producers using whole-genome sequencing (WGS). A total of 36 *Acinetobacter* isolates collected at three hospitals in Ghana between 2016 and 2017 were analyzed. MICs were determined by commercial antibiotic plates. *Acinetobacter baumannii* MLST was determined using the Pasteur scheme. WGS of OXA-carbapenemase producers was performed using short- and long-read sequencing strategies. The resistance rate was highest for trimethoprim/sulfamethoxazole (*n* = 22; 61%). Six (16.7%) and eight (22.2%) isolates were resistant to ceftazidime and colistin, respectively. Two (5.6%) isolates were resistant and one (2.8%) isolate had intermediate sensitivity to three carbapenems. Fifteen STs were identified in 24 *A. baumannii* isolates including six new STs (ST1467 ∼ ST1472). ST78 was the predominant (*n* = 6) followed by ST1469 (*n* = 3). Four carbapenemase-producing *A. baumannii* isolates also were identified. Isogenic ST103 isolates Ab-B004d-c and Ab-D10a-a harbored *bla*_OXA–__23_ within Tn*2007* on identical plasmids, pAb-B004d-c_3, and pAb-D10a-a_3. ST1472 isolate Ab-C102 and ST107 isolate Ab-C63 carried *bla*_OXA–__58_ and *bla*_OXA–__420_, a rare *bla*_OXA–__58_ variant, respectively, within novel genetic contexts. Our results show that *A. baumannii* isolates of diverse and unique genotypes, including OXA-carbapenemase producers, are circulating in Ghana highlighting the need for a wider surveillance of antimicrobial resistance.

## Introduction

*Acinetobacter* species are important pathogens, responsible for healthcare-related infections. *Acinetobacter baumannii* is the most clinically prevalent species, but the clinical significance of *A. pittii* and *A. nosocomialis* is also increasing (Adams et al., 2008; Chuang et al., 2011; Nemec et al., 2011; Doi et al., 2015). As *Acinetobacter* spp. have intrinsic resistance to several antibiotic classes and can acquire resistance determinants, carbapenems are an optimal treatment option for infections caused by these bacteria. However, carbapenem resistance is rising worldwide, leaving very limited antimicrobial treatment options (Dijkshoorn et al., 2007; Hamidian and Nigro, 2019). Therefore, carbapenem-resistant *Acinetobacter* (CRA) is globally recognized as an urgent threat (World Health Organization, 2017; Centers for Disease Control and Prevention, 2019).

Carbapenem resistance in *Acinetobacter* is mainly caused by the production of carbapenem-hydrolyzing class D β-lactamases (CHDLs) including OXA-23-like, OXA-24-like and OXA-58-like enzymes, or by the production of Ambler class B metallo-β-lactamases (MBLs) such as NDM-, IMP- and VIM-type enzymes (Potron et al., 2015). Until now, only NDM-1-producing *A. baumannii* has been reported in Accra, the capital of Ghana (Codjoe et al., 2019). In *A. baumannii*, overexpression of the intrinsic *bla*_OXA–__51_-like gene mediated by the insertion sequence IS*Aba1* upstream also confers resistance to carbapenems (Wong et al., 2019). Changes in outer membrane proteins and overexpression of efflux pumps are also associated with carbapenem resistance (Poirel and Nordmann, 2006a; Coyne et al., 2011).

Surveillance of CRA prevalence, associated genotypes, and elucidating the mechanisms underlying resistance are crucial as they enable us to understand how these bacteria disseminate and how best to control their spread. However, due to the poor antimicrobial resistance surveillance in Ghana, data on CRA is limited. Moreover, no data is available yet for CHDL-producing *Acinetobacter* and their genotypic characteristics.

Our aim was to investigate the antibiotic resistance profile and the genotypic characteristics of clinical isolates of *Acinetobacter* spp. collected at three regional hospitals in Ghana. Furthermore, we also characterize OXA-carbapenemase producers using both short- and long-read whole-genome sequencing (WGS).

## Materials and Methods

### Bacterial Isolates and Identification

A total of 36 non-redundant *Acinetobacter* clinical isolates were recovered from different patients at three hospitals in Ghana (Tamale Teaching Hospital, *n* = 3; Cape Coast Teaching Hospital, *n* = 2; and Effia Nkwanta Regional Hospital, *n* = 31) between 2016 and 2017. Specimen types included urine (*n* = 12), sputum (*n* = 8), wound (*n* = 3), high vaginal swabs (*n* = 2), blood (*n* = 1), semen (*n* = 1) and cerebrospinal fluid (*n* = 1). For the remaining six samples, specimen types were not found from the medical records. Microbial identification was performed using the MALDI Biotyper (Bruker Daltonics, Karlsruhe, Germany). *Acinetobacter baumannii* complex (ABC) isolates were further characterized to species level using *gyrB* multiplex PCR method as previously described (Higgins et al., 2010).

### Antibiotic Susceptibility Testing

The MICs of 15 antibiotics including meropenem, colistin, and minocycline were determined by broth microdilution using the commercial microplates DP34 and DP35 (Eiken Chemical Co., Tokyo, Japan). Results were interpreted according to the Clinical and Laboratory Standards Institute guideline document M100-S27 (Clinical and Laboratory Standards Institute, 2017).

### Screening for Carbapenemase Genes

DNA was extracted using the Cica Geneus^TM^ DNA Extraction reagent (Kanto Chemical Co., Tokyo, Japan) and subsequently screened for common CHDLs such as *bla*_OXA–__23_-like, *bla*_OXA–__24_-like, *bla*_OXA–__58_-like, and *bla*_OXA–__235_-like, as well as the *A. baumannii* native β-lactamase *bla*_OXA–__51__–_like, using multiplex PCR, as previously described (Woodford et al., 2006; Higgins et al., 2013). Screenings for other carbapenemase genes including *bla*_OXA–__48_, *bla*_NDM_, *bla*_KPC_, *bla*_IMP_, *bla*_VIM_, and *bla*_GES_ were also conducted for all isolates (Dallenne et al., 2010; Wachino et al., 2011).

### Multilocus Sequence Typing

Multilocus sequence typing (MLST) was performed for all confirmed *A. baumannii* isolates using the Pasteur scheme. New allele types and new sequence types (STs) were submitted to PubMLST^1^ and assigned new numbers. Genetic relatedness of STs was analyzed and visualized using PHYLOVIZ 2.0 goBURST Full MST analysis (Nascimento et al., 2017).

### Whole-Genome Sequencing

Short- and long-read WGS was conducted in four CHDL-producing isolates to determine their complete chromosome and plasmid sequences with high accuracy. DNA was extracted with the NucleoSpin tissue kit (Macherey-Nagel, Düren, Germany) for short-read sequencing; in one of the isolates the DNA library was prepared with the Nextera DNA Flex library prep kit and sequenced with Illumina Miseq (Illumina, San Diego, CA, United States); GIEasy FS PCR-Free DNA Library Prep Set was used for the remaining three isolates and sequenced with MGI DNBSEQ (MGI Tech Co., Shenzhen, China). Nanopore MinION (Oxford Nanopore Technologies, Oxford, United Kingdom) was used for long-read sequencing; DNA was extracted with the MagAttract HMW DNA Kit (Qiagen, Hildon, Germany); multiplexed DNA libraries were prepared for all four isolates using the Native Barcoding Kit EXP-NBD104 and the ligation sequence Kit SQK-LSK109; and sequencing was performed on a single R9.4.1 flow cell. Low-quality reads (MinION *Q* < 10; DNBSEQ *Q* < 32; and MiSeq *Q* < 30) and short reads (MinION length <1,000 bp; DNBSEQ and Miseq length <10 bp) were filtered out.

### Bioinformatics Analysis

Hybrid *de novo* genome assembly for WGS data was done with Unicycler v0.4.8 and Flye v2.6 using both short and long reads. Sequence annotation was performed using the online system RAST and BLAST searches. Whole-genome SNP typing was done for four CHDL producers using PathoBacTyper^2^. Resfinder v3.2 was used to detect acquired resistance genes (Zankari et al., 2012). Insertion sequences were identified using ISfinder^3^. Plasmid structures and genetic contexts of carbapenemase genes were compared and visualized using Easyfig v2.2.2 (Sullivan et al., 2011).

### Phylogenetic Analysis

Forty-five *bla*_OXA–__58_ plasmids and one *bla*_OXA–__420_ plasmid in the repository of PLSDB v.2020_03_04 (Galata et al., 2019), as well as two plasmids from this study (pAbC102_1 and pAbC63_1) were used for phylogenetic analysis. Sequence alignment of plasmids was performed using CLC genomic workbench 20 (CLC bio, Aarhus, Denmark). A maximum parsimony tree with 1,000 bootstrap replicates was generated using MEGA X 10.1 (Kumar et al., 2018).

### Accession Numbers

Complete chromosomal and plasmid sequences were deposited in GenBank under Bioproject PRJNA473419.

## Results

### Phenotypic Characterization of *Acinetobacter* Isolates

Thirty-five out of the 36 isolates belonged to ABC, while the other isolate was identified as *A. haemolyticus.* Among all ABCs, 24 isolates (68.6%) were characterized as *A. baumannii* followed by seven *A. nosocomialis* (20%) and three *A. pittii* (8.6%). The species for the remaining one ABC isolate (identified using MALDI Biotyper) was not determined due to conflicting results from the *gyrB* multiplex PCR; this isolate was negative for the *bla*_OXA–__51_-like.

Resistance to trimethoprim/sulfamethoxazole was the most prevalent (*n* = 22; 61%), followed by resistance to piperacillin (*n* = 14; 38.9%), and tobramycin (*n* = 13; 36.1%) (Table 1). Six (16.7%) isolates were resistant to ceftazidime and eight (22.2%) were resistant to colistin. Two (5.6%) were resistant and one (2.8%) had intermediate susceptibility to three carbapenems. Resistance to amikacin and minocycline was the least frequent (*n* = 1; 2.8% for each). Resistance to all antibiotics, except for amikacin and colistin, were higher among *A. baumannii* than non-*baumannii Acinetobacter* isolates.

**TABLE 1:** Antibiotic resistance profile of 36 *Acinetobacter* isolates.

**Antibiotic**	**All isolates (*n* = 36)**				***A. baumannii* isolates (*n* = 24)**				**Non-*baumannii Acinetobacter* isolates (*n* = 12)**			
	**MIC (μg/ml)**			**%R^a^**	**MIC (μg/ml)**			**%R^a^**	**MIC (μg/ml)**			**%R^a^**
	**Range**	**MIC_50_**	**MIC_90_**		**Range**	**MIC_50_**	**MIC_90_**		**Range**	**MIC_50_**	**MIC_90_**	
Piperacillin	8 to >64	32	>64	38.9	8 to >64	>64	>64	50	8 to >64	32	>64	16.7
Ampicillin-sulbactam	1/2 to >8/16	1/2	4/8	5.6	1/2 to >8/16	2/4	8/16	8.3	1/2 to 4/8	1/2	2/4	0
Piperacillin-tazobactam	≤4/16 to >4/64	≤4/16	>4/64	22.2	≤4/16 to >4/64	4/32	>4/64	29.2	≤4/16 to >4/64	≤4/16	4/32	8.3
Ceftazidime	2 to >16	4	>16	16.7	4 to >16	4	>16	20.8	2 to >16	4	16	8.3
Cefepime	1 to >16	4	>16	13.9	2 to >16	4	>16	16.7	1 to >16	2	16	8.3
Imipenem	≤0.25 to >8	≤0.25	1	5.6	≤0.25 to >8	≤0.25	4	8.3	≤0.25 to 1	≤0.25	≤0.25	0
Meropenem	≤0.25 to >8	0.5	2	5.6	≤0.25 to >8	0.5	4	8.3	≤0.25 to 2	≤0.25	1	0
Doripenem	≤0.25 to >8	≤0.25	1	5.6	≤0.25 to >8	0.5	4	8.3	≤0.25 to 1	≤0.25	1	0
Gentamycin	0.5 to >8	0.5	>8	33.3	0.5 to >8	>8	>8	50	0.5 to 2	0.5	2	0
Tobramycin	0.5 to >8	1	>8	36.1	0.5 to >8	8	>8	50	0.5 to >8	0.5	2	8.3
Amikacin	≤1 to >32	2	4	2.8	2 to 16	2	4	0	≤1 to >32	2	8	1.3
Minocycline	≤0.25 to >8	0.5	2	2.8	≤0.25 to >8	0.5	4	4.2	≤0.25 to 4	≤0.25	1	0
Colistin	≤2 to 8	≤2	4	22.2	≤2 to 4	≤2	4	12.5	≤2 to >8	≤2	8	41.7
Ciprofloxacin	0.12 to >2	0.5	>2	33.3	0.12 to >2	>2	>2	50	0.12 to 1	0.25	1	0
Levofloxacin	≤1 to >4	≤1	>4	16.7	≤1 to >4	4	>4	25	≤1	≤1	≤1	0
Trimethoprim-sulfamethoxazole	≤19/1 to >38/2	>38/2	>38/2	61.1	≤19/1 to >38/2	>38/2	>38/2	66.7	≤19/1 to >38/2	>38/2	>38/2	50

*^*a*^Resistant.*

### Detection of Carbapenemase Producers

Two *A. baumannii* isolates, Ab-D10a-a and Ab-B004d-c, belonging to ST103, and harboring *bla*_OXA–__23_ were identified. The allelic variant of *bla*_OXA–__51_ was *bla*_OXA–__70_ for both isolates. In addition, *A. baumannii* isolates, Ab-C102A and Ab-C63, belonging to the novel ST1472 and ST107 with *bla*_OXA–__51_ variants of *bla*_OXA–__699_ and *bla*_OXA–__51_ respectively, were also identified and found to harbor CHDLs, *bla*_OXA–__58_ and *bla*_OXA–__420_. All four isolates were collected at the Effia Nkwanta Regional Hospital. Other carbapenemase genes, such as MBL genes were not detected by conventional PCR.

### Genetic Diversity of *Acinetobacter baumannii* Isolates

**FIGURE 1:**
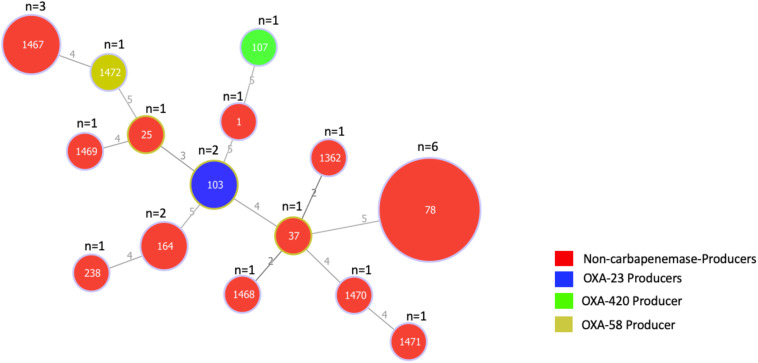
Minimum spanning tree based on the allelic profiles of 15 STs identified in 24 *A. baumannii* isolates. The number of isolates for each ST is shown at the top of each node. Node sizes are proportional to the number of isolates for each ST and numbers on connecting lines indicate the number of locus variants determined by pair-wise comparisons. Colors indicate whether isolates are carbapenemase producers and if so, the type of carbapenemase they produce.

The genetic relatedness of the 24 *A. baumannii* isolates was assessed using the Pasteur MLST scheme. Isolates were assigned into 15 different STs, six of which were novel STs (ST1467, ST1468, ST1469, ST1470, ST1471, and ST1472). Minimum spanning tree analysis revealed that all STs had no genetic relationship with each other (Figure 1). ST78 (*n* = 6; 25%) was the most predominant, followed by the novel ST1467 (*n* = 3; 12.5%).

### Genotypic Characterization of Ab-B004d-c, Ab-D10a-a, and *bla*_OXA–__23_ Genetic Context

**FIGURE 2:**
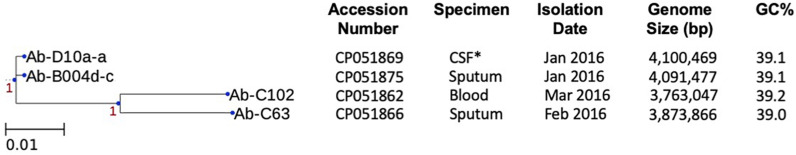
Whole-genome SNP analysis of chromosomal sequences of four CHDL-producing isolates and their characteristics. Scale bar indicates nucleotide substitutions per site (1 SNP per 100 sites). Confidence levels are shown close to the branches. *CSF, cerebral spinal fluid.

Two OXA-23-producing ST103 isolates, Ab-B004d-c, and Ab-D10a-a, were recovered from sputum and cerebrospinal fluid samples of different patients in January 2016 (Figure 2). Both isolates were resistant to most antibiotics, including carbapenems, but were fully susceptible to colistin (Table 2). Over 4 Gb short-read sequences and about 1.8 Gb long-read sequences were obtained for each isolate after quality filtering (Supplementary Table 1). Using *de novo* hybrid assembly, two circular chromosomes with 4,091,477 and 4,100,469 bp were generated for Ab-B004d-c and Ab-D10a-a, respectively (Supplementary Table 2). Both isolates were isogenic, differing by only 10 SNPs (Figure 2). Four pairs of identical plasmids ranging in size from 2,697–48,239 bp also were generated for both isolates (Supplementary Table 2). Ab-D10a-a carried an extra 6,619 bp plasmid (Supplementary Table 2). The chromosomes of both isolates carried the aminoglycoside resistance gene *aac*(3)-IIa, the sulphonamide resistance gene *sul*1, the intrinsic β-lactamase gene *bla*_OXA–__70_, the extended-spectrum β-lactamase (ESBL) gene *bla*_CTX–M–__15_, and the intrinsic AmpC gene *bla*_ADC–__203_. No insertion sequences were detected upstream of *bla*_OXA–__70_ and *bla*_ADC–__203_. The trimethoprim resistance gene *dfr*A1, the aminoglycoside resistance gene *aac*(3)-IId, the tetracycline resistance gene *tet*(39), and macrolide resistance genes *msr*(E) and *mph*(E) were found on two identical plasmids with 48,239 bp (pAb-B004d-c_1 and pAb-D10a-a_1) in both isolates.

**TABLE 2:** Genotypic and phenotypic antibiotic resistance profiles of four CHDL-producing *A. baumannii* isolates.

**Isolates**	**CHDL type**	**Chromosome/plasmid**	**Resistance genes**	**MIC (mg/L)**															
				**PIPC**	**S/A**	**TAZ/PIPC**	**CAZ**	**CFPM**	**IPM**	**MEPM**	**DRPM**	**GM**	**TOB**	**AMK**	**MINO**	**CL**	**CPFX**	**LVFX**	**ST**
Ab-B004d-c	OXA-23	Chromosome	*bla*ADC-66, *bla*OXA-70, *sul*1, *ant*(3″)-IIa, *aac*(3)-IIa and *bla*CTX-M-15	>64	>8/16	>4/64	>16	>16	>8	>8	>8	>8	>8	8	0.5	≤2	>2	>4	>38/2
		Plasmid	*dfr*A1, *mph*(E), *msr*(E),*tet*(39), *aac*(3)-IId and *bla*OXA-23																
Ab-D10a-a	OXA-23	Chromosome	*bla*ADC-66,*bla*OXA-70, *sul*1, *ant*(3″)-IIa, *aac*(3)-IIa and *bla*CTX-M-15	>64	>8/16	>4/64	>16	>16	>8	>8	>8	>8	>8	16	0.5	≤2	>2	>4	>38/2
		Plasmid	*dfr*A1, *mph*(E), *msr*(E), *tet*(39), *aac*(3)-IId and *bla*OXA-23																
Ab-C102	OXA-58	Chromosome	*ant*(3″)-IIa, *bla*OXA-699 and *bla*ADC-32	64	1/2	4/32	8	4	1	1	0.5	0.5	2	2	≤0.25	≤2	0.5	≤1	>38/2
		Plasmid	*aph*(6)-Id, *aph*(3″)-Ib, *sul*2, *aph*(3′)-Ia, *flo*R and *bla*OXA-58																
Ab-C63	OXA-420	Chromosome	*bla*ADC-87, *bla*OXA-51 and *ant*(3″)-IIa	>64	8/16	>4/64	4	8	4	4	4	>8	>8	2	0.5	4	>2	>4	>38/2
		Plasmid	*mph*(E), *msr*(E), *tet*(39), *dfr*A20, *sul*2, *aph*(6)-Id, *aph*(3″)-Ib, *bla*OXA-420, *ant*(2″)-Ia and *aph*(3’)-Ia																

*Abbreviations: PIPC, piperacillin; S/A, ampicillin-sulbactam; TAZ/PIPC, piperacillin-tazobactam; CAZ, ceftazidime; CFPM, cefepime; IPM, imipenem; MEPM, meropenem; DRPM, doripenem; GM, gentamycin; TOB, tobramycin; AMK, amikacin; MINO, minocycline; CL, colistin, CPFX, ciprofloxacin; LVFX, levofloxacin; ST, trimethoprim-sulfamethoxazole.*

**FIGURE 3:**
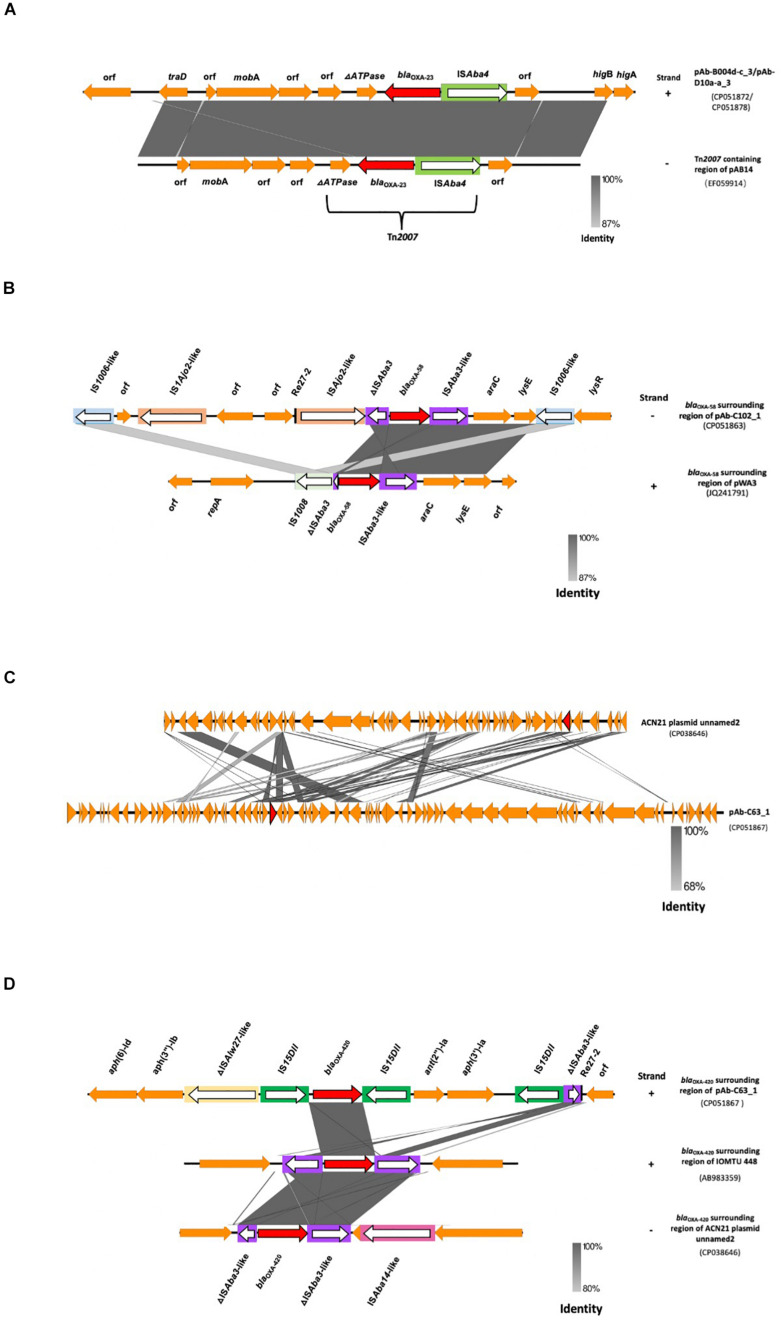
Genetic contexts of CHDLs in *A. baumannii* from Ghana. **(A)** Linearized whole plasmid sequence of *bla*_OXA–__23_-containing plasmids pAb-B004d-c_3 and pAb-D10a**-**a_3, compared to the Tn*2007*-containing region of pAB14. **(B)** *bla*_OXA–__58_-surrounding region of pAb-C102_1 compared with that of pWA3. **(C)** Linearized whole plasmid backbone structure of pAb-C63_1 compared with that of ACN21 plasmid unnamed2. **(D)** *bla*_OXA–__420_-surrounding region of pAb-C102_1 compared with that of IOMTU 448 and ACN21 plasmid unnamed2. Carbapenemase genes are represented by red arrows, and other coding sequences are represented in orange. IS elements are inside rectangular boxes. The direction of transposase genes is indicated by white arrows. Same or highly similar IS elements are depicted in the same colors. Re-27 sequences are represented by vertical black bars. Positive and reverse strands are labeled at the right end of each sequence with the symbols “+” and “–”, respectively.

The *bla*_OXA–__23_ gene was present on two identical 8,215 bp plasmids (pAb-B004d-c_3 and pAb-D10a-a_3) within Tn*2007*, and flanked upstream by IS*Aba4* and downstream by a truncated ATPase gene (Figure 3A). No other resistance genes were detected in these plasmids. Annotation results and BLAST searches for ORFs failed to identify any replication origin protein (Rep) gene in these plasmids. The linearized sequences of pAb-B004d-c_3 and pAb-D10a-a_3 were compared with the *bla*_OXA–__23_*-*containing region of pAB14 from *A. baumannii* (accession number: EF059914). Results showed that the genetic structure of Tn*2007* was highly identical in pAb-B004d-c_3, pAb-D10a-a_3 and pAB14. In addition, a *higBA* toxin-antitoxin locus was found downstream of Tn*2007*.

### Genotypic Characterization of Ab-C102 and *bla*_OXA–__58_ Genetic Context

The OXA-58-producing ST1472 isolate Ab-C102 was recovered from the blood of a patient. Ab-C102 was susceptible to almost all antibiotics but displayed a low meropenem MIC (1 mg/L) (Table 2).

Similar to the isolates described before, more than 4 Gb of short-read and near 1 Gb of long-read sequences were obtained by WGS for the isolate Ab-C102, after quality filtering (Supplementary Table 1). A circular chromosome with 3,763,047 bp and three circular plasmids with 19,853, 67,097, and 90,089 bp were generated by hybrid *de novo* genome assembly (Supplementary Table 2). A novel variant of the intrinsic *bla*_OXA–__65_ gene with three synonymous nucleotide substitutions, and an intrinsic *bla*_ADC–__32_ gene were identified on the chromosome. No insertion sequences were detected upstream of these genes. The carbapenemase gene *bla*_OXA–__58_ along with other resistance genes such as *aph*(6)-Id were identified on a 90,089 bp plasmid (pAb-C102_1; Table 2).

**FIGURE 4:**
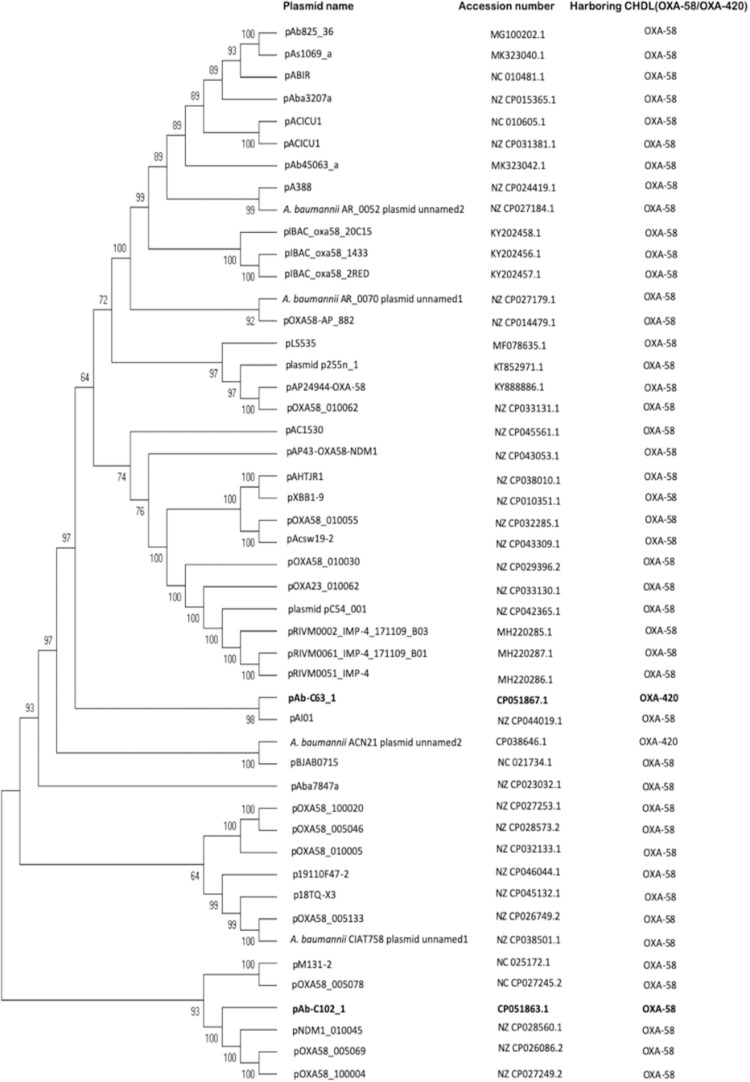
Maximum parsimony tree of OXA-58- and OXA-420-containing plasmids. Two plasmids from the current study (highlighted in bold), 45 OXA-58-producing plasmids and one OXA-420-producing plasmid listed in PLSDB database were included. Bootstrap values are shown at each node.

This plasmid encodes a RepB family plasmid Rep that share 100% identity with its counterpart in *A. baumannii* WB103 (accession number: AZM37906). Phylogenetic analysis showed that the genetically closest plasmid to pAb-C102_1 was pNDM-1_010045, with which it share 46% query cover and 97.25% identity (Figure 4). Interestingly, a BLAST search found that the full plasmid sequence of pAb-C102_1 had the highest homology with the draft WGS data of WB103, sharing 88% of query cover and 99.88% of identity.

The genetic environment of *bla*_OXA–__58_ in pAb-C102_1 was comparable to that in pWA3 (accession number: JQ241791), with a similar genetic structure (Figure 3B). In both plasmids, the same IS*Aba3*-like-*ara*C1-*lys*E structure was found downstream of *bla*_OXA–__58_ and the same three nucleotide substitutions were present in both IS*Aba3*-like. The IS*Aba3*-like-*ara*C1-*lys*E was followed by one IS*1006*-like copy (one nucleotide substitution compared to IS*1006*) and the *lys*R gene. Upstream of *bla*_OXA–__58_, a 427 bp truncated form of IS*Aba3* was interrupted by a putative novel IS*Ajo2*-like sequence. This contrasted with pWA3 in which a 121 bp truncated form of IS*Aba3* is interrupted by IS*1008*. A Re27-2 region, which is a p*dif* site targeted by the XerC/XerD-like site-specific recombinases, was found 13 bp upstream of the IS*Ajo2*-like sequence. A second copy of IS*Ajo2*-like and IS*1006*-like (three nucleotide substitutions compared with IS*1006*) was found upstream of the Re27-2 region. Neither a second copy of Re27-2 nor Re27-1 (single nucleotide variant of Re27-2) were found downstream of *bla*_OXA–__58_.

### Genotypic Characterization of Ab-C63 and *bla*_OXA–__420_ Genetic Context

Ab-C63 was an isolate recovered from a sputum sample that belonged to ST107 and produced OXA-420, a rare CHDL of the OXA-58 family. This isolate had intermediate susceptibility to carbapenems and was resistant to aminoglycosides, fluoroquinolones and colistin (Table 2).

A 3,873,864 bp circular chromosome and two plasmids with 81,353 and 10,662 bp were generated by hybrid assembly using quality-filtered ∼350 Mb short-read and ∼1.2 Gb long-read sequences (Supplementary Tables 1 and 2). The intrinsic β-lactamase genes *bla*_ADC–__87_ and *bla*_OXA–__51_, as well as the aminoglycoside resistance gene *ant*(3″)-IIa were found on the chromosome. The *bla*_OXA–__420_ gene and other resistance genes such as *mph*(E) were located on a plasmid with 81,353 bp (pAb-C63_1; Table 2). This plasmid harbored a novel gene encoding a RepB family plasmid Rep which shared the highest similarity (99.7% identity) with proteins from *Acinetobacter* spp. and *A. brisouii* (accession numbers: WP_004761033, WP_151711075).

Phylogenetic analysis (Figure 4) showed pAB-C63_1 to be genetically distinct from the *bla*_OXA–__420_-containing *A. baumannii* ACN21 plasmid unnamed2 (NZ_CP038646), collected in India, but genetically close to the *bla*_OXA–__58_-containing pAI01 (NZ_CP044019), with which it shares 42% query cover and 95% identity. Plasmid backbone structure comparisons also revealed that pAb-C63_1 had a novel backbone compared with ACN21 plasmid unnamed2 (Figure 3C). Moreover, a completely distinct genetic context was found surrounding *bla*_OXA–__420_ in pAbC63_1, in which the gene was flanked by two copies of IS*15DII*, in contrast with ACN21 plasmid unnamed2 and *A. baumannii* strain IOMTU in which *bla*_OXA–__420_ is flanked by IS*Aba3-*like and truncated IS*Aba3-*like elements (Figure 3D). In addition, a third copy of IS*15DII* was found downstream, interrupting an IS*Aba3* sequence. The resistance genes *ant*(2″)-Ia and *aph*(3′)-Ia were flanked by the second and third copies of IS*15DII*. The Re27-2 sequence was detected 65 bp downstream of the truncated IS*Aba3.* Moreover, a novel insertion sequence, a ΔIS*Alw27*-like element (coverage = 1268/1282; identities = 1213/1268), was detected upstream of the first copy of IS*15DII*.

## Discussion

Carbapenem resistance in *Acinetobacter* spp. is a major health threat worldwide. Most of the studies on CRA in Africa were conducted in Northern or Southern regions (Manenzhe et al., 2015), while the current situation in sub-Saharan Africa may be underestimated given the limited laboratory and surveillance capacity. Here, we showed that *Acinetobacter* species, predominantly *A. baumannii*, are disseminating in Ghanaian healthcare facilities. *A. nosocomialis* and *A. pittii* (previously known as *Acinetobacter* genomics species 13UT and 3, respectively) were also prevalent. The proportion of these species is parallel to those causing nosocomial bloodstream infections in the United States (Wisplinghoff et al., 2012). Conventional identification methods can not distinguish between ABCs, resulting in misidentification of some species as *A. baumannii* (Wisplinghoff et al., 2012). Although, *gyrB* multiplex PCR has been reported as one of the most highly sensitive methods distinguishing species within ABCs (Lee et al., 2014), the conflicting result that we obtained for an ABC isolate using this method has proven the difficulty associated with species identification among ABCs. Multidrug resistance, including to carbapenems, was detected in the *Acinetobacter* isolates. However, carbapenem resistance was less prevalent (5.6%), in our study (primarily conducted in the Western region of Ghana) compared with a prior study conducted in the country’s capital, Accra, in which seven out of nine *A. baumannii* isolates from burn wounds were resistant to meropenem (Forson et al., 2017). Higher carbapenem resistance, 51.5 and 10%, have been reported in the West African countries of Nigeria and Sierra Leone, respectively (Nsofor and Chekwube, 2017; Lakoh et al., 2020). Such discrepancy suggests that CRA dissemination varies by region, and may be attributed to the variability in antibiotic choice, infection control, and prevention practices. Moreover, the use of carbapenems in clinical setting in Ghana has been reported as low, and its unavailability as an essential drug on the Ghanaian national health insurance scheme may be associated with low prescription (Labi et al., 2018). We speculate this low carbapenems usage is correlated with low resistance selective pressure which may explain the observed lower carbapenem resistance prevalence in our study. The 22.2% prevalence of colistin resistance among all *Acinetobacter* isolates as well as 12.5% among *A. baumannii* isolates in this study are high in comparison with other countries where the resistance prevalence was mostly at single digit levels for *A. baumannii* (Pormohammad et al., 2020). Such high resistance is alarming especially since colistin has become one of the last-resort antibiotics used for multidrug-resistant gram-negative bacterial infections. The acquired resistance mediated by plasmid originated *mcr* gene resulted in the global spread of the colistin resistance among *Enterobacteriaceae* (Wang et al., 2018; Wise et al., 2018). In *A. baumannii*, colistin resistance is not associated with the *mcr* gene, rather caused by the overexpressed PetN transferases mediated by two pathways, overexpressed *pmrC* from mutations in *pmrCAB* operon and overexpressed *pmrC* homolog *eptA* from the insertion of IS*Aba1* on the upstream (Trebosc et al., 2019). Further investigation remains warranted to elucidate the resistance mechanisms involved in non-*baumannii Acinetobacter* isolates.

Several global clones (GC) of *A. baumannii* have emerged in the last decades with GC2, represented by ST2, being the most widespread, followed by GC1, represented by ST1. Multidrug resistance and carbapenemase production are mostly associated with the spread of these specific clones (Hamidian and Nigro, 2019). Here, we showed that *A. baumannii* isolates recovered in Ghana have high genetic diversity, associated with 15 STs among which six are novel. The predominant GC2 was absent and only a single isolate belonged to GC1. ST78, also known as the “Italian clone” (Carretto et al., 2011), was the predominant sequence type detected in Ghana. However, neither ST1 nor ST78 isolates were associated with carbapenemase production. The CHDL producers found belong to ST103, ST107 and ST1472; Both of ST103, and ST107 were uncommon, while ST1472 is a novel type. OXA-23 production is the leading carbapenem resistance mechanism in *A. baumannii*, mostly associated with GC2 and GC1 (Hamidian and Nigro, 2019). Although ST103 strains were associated with NDM-2-producing isolates in Egypt, UAE, and Palestine (Kaase et al., 2011; Ghazawi et al., 2012; Sjölander et al., 2014), and an OXA-235-producing strain was reported in Australia (Hamidian et al., 2017), to the best of our knowledge this is the first study to identify ST103 associated with OXA-23 production (isolates Ab-B004d-c and Ab-D10a-a). Based on the occurrence of OXA-58 carbapenemase in several *Acinetobacter* species and in *Proteus mirabilis* (Fu et al., 2014; Feng et al., 2016; Lange et al., 2017; Matos et al., 2019), it has been suggested that horizontal gene transfer rather than clonal spread is the main mechanism of *bla*_OXA–__58_ dissemination. This may also explain the detection of a novel sequence type, ST1472 (isolate Ab-C102). ST107 isolate Ab-C63 carried *bla*_OXA–__420_, a rare variant of *bla*_OXA–__58_ that was first described in *A. baumannii* ST623 and ST32 clinical isolates in Nepal (Shrestha et al., 2015). Ab-C63 was genetically distinct from the Nepalese isolates and other isolates identified in France and Brazil, which had the same ST and produced different CHDLs including OXA-24, OXA-143 and OXA-231 (Jeannot et al., 2014; Rodrigues-Costa et al., 2019).

We identified two isogenic OXA-23 producers, Ab-B004d-c and Ab-D10a-a. These two isolates were recovered from different patients in the same hospital, indicating a possible nosocomial outbreak. The ESBL gene *bla*_CTX–M–__15_ was also found on the chromosome of both isolates. Third-generation cephalosporin resistance in *A. baumannii* results mainly from the production of ESBLs of PER, GES and VEB types, or the overexpression of intrinsic AmpC β-lactamases owing to the upstream insertion of specific sequences such as IS*Aba1* (Hamidian and Hall, 2013; Potron et al., 2015). The ESBL gene *bla*_CTX–M–__15_ is the main mechanism of resistance to third-generation cephalosporins in *Enterobacteriaceae*, but not a common one in *Acinetobacter*. There are only a few report of its occurrence in *A. baumannii* isolates from India, Haiti, and Brazil (Shakil and Khan, 2010; Potron et al., 2011; Zago et al., 2016). Therefore, by detecting *bla*_CTX–M–__15_ in isolates from Ghana, our study provides evidence of its wider spread among *Acinetobacter* species.

The *bla*_OXA–__23_ gene is the most common carbapenemase gene in *A. baumannii* and is usually mobilized by Tn*2006*, Tn*2007*, Tn*2008* (including its variants), Tn*2009*, Tn*6549*, and AbaR4 into the chromosome and plasmids. Tn*2006* is the most abundant of these mobile elements (Hamidian and Nigro, 2019). Tn*2007*, which was identified in plasmids pAb-B004d-c_3 and pAb-D10a-a_3 is relatively uncommon with no plasmids listed on PLSDB and only one in GenBank (pAB14) carrying it. However, its detection has been reported in isolates from Algeria, France, Spain, China, and Tanzania (Corvec et al., 2007; Guerrero-Lozano et al., 2015; Wang et al., 2015; Kumburu et al., 2019). The identification of Tn*2007* in samples from Ghana, within a plasmid that share a high level of sequence identity with pAB14 (identified in Algeria and Tanzania) strongly supports the importance of plasmids in the spread of carbapenemase genes, especially in Africa.

The *bla*_OXA–__58_-containing plasmid pAb-C102_1 (isolate Ab-C102) share the same Rep as well as the highest query cover and sequence homology with the draft genome of *A. baumannii* WB103, an isolate recovered from hospital wastewaters in Singapore and that also carry *bla*_OXA–__58_ (Chen et al., 2019). It was not possible to compare the genetic structure between the two isolates because WB103 plasmid sequences are not assembled, but the findings suggest that pAb-C102_1 likely derived from a plasmid from WB103.

Diverse genetic environments are described for *bla*_OXA–__58_. The gene is commonly embedded in structures like IS*Aba2*:IS*Aba3*-*bla*_OXA–__58_-IS*Aba3* and IS*6* family element-ΔIS*Aba3*-*bla*_OXA–__58_-IS*Aba3*. Usually, these structures are accompanied downstream by *ara*C1 and *lys*E (Poirel and Nordmann, 2006b; Fu et al., 2014; Cameranesi et al., 2018); the former is involved in *bla*_OXA–__58_ regulation (Fondi et al., 2010). Mobilization and acquisition of *bla*_OXA–__58_ is driven by site-specific recombination at XerC/D-like sites (including mediation by Re27) rather than through its surrounding IS elements, leading to diverse structure arrangements (Poirel and Nordmann, 2006b; Cameranesi et al., 2018). We observed a similar pattern in pAb-C102_1, in which an IS*Aba3* element interrupted by a novel IS*Ajo2*-like sequence was followed by IS*Aba3*-like-*ara*C1-lysE, a structure that commonly surrounds *bla*_OXA–__58_. In addition, a Re27-2 site was found next to the novel IS*Ajo2*-like element. These results indicate that *bla*_OXA–__58_ was acquired via a similar XerC/D-like site-specific recombination in pAb-C102_1, and that the second copy of the gene created in the process may have been lost. Moreover, different arrangements in insertion sequences upstream of *bla*_OXA–__58_ were associated with its overexpression; compared with IS*Aba3* this resulted in a stronger promoter leading to higher carbapenem resistance levels (Chen et al., 2008, 2010; Fu et al., 2014). It is unclear whether the low carbapenem MIC levels in Ab-C102 is a consequence of IS*Ajo2*-like-ΔIS*Aba3* arrangement and a relatively weak *bla*_OXA–__58_ promoter. Further investigations are needed to clarify this issue.

The gene *bla*_OXA–__420_ has three nucleotide substitutions and a single amino acid substitution compared with *bla*_OXA–__58_, and was first identified in 16 *A. baumannii* isolates from Nepal (Shrestha et al., 2015). Our BLAST search identified *A. baumannii* strain ACN21, isolated from India, that also carry *bla*_OXA–__420_, indicating that this specific variant is mostly spread in South Asia. To the best of our knowledge this study is the first to detect a *bla*_OXA–__420_-carrying isolate outside of Asia. Isolate Ab-C63 is not genetically related to earlier isolates from Nepal and India. Moreover, the genetic context of *bla*_OXA–__420_ in pAb-C63_1 is novel while those from India and Nepal are similar. The presence of a Re27-2 sequence, commonly associated with *bla*_OXA–__58_ acquisition, near *bla*_OXA–__420_, suggests that this resistance gene also was acquired by XerC/D-mediated site-specific recombination in Ab-C63.

One limitation of our study is the limited sample size. Isolates were collected at three hospitals only, and the number of isolates obtained from two of the hospitals was extremely low. Hence, the results may not reflect the whole situation in Ghana.

In this study, we have demonstrated that the prevalence of carbapenem resistance in Ghana is low and that there is a high level of genetic diversity among clinical isolates of *Acinetobacter*. Furthermore, we characterized, for the first time in Ghana, OXA- 23-, OXA-58- and OXA-420-producing *A. baumannii* isolated belonging to ST103, ST1472 and ST107, respectively. By combining short-read and long-read WGS we revealed that *bla*_OXA–__23_ was within Tn*2007* in two plasmids (pAb-B004d-c_3 and pAb-D10a-a_3) and identified novel genetic structures surrounding *bla*_OXA–__58_ and *bla*_OXA–__420_ (novel plasmids pAb-C102_1 and pAb-C63_1, respectively). Altogether, our results show that genetically diverse and unique *A. baumannii* isolates, including OXA-carbapenemase producers, are circulating in Ghana, measures to strengthen and expand surveillance of antimicrobial resistance worldwide remains essential.

## Data Availability Statement

The datasets presented in this study can be found in online repositories. The names of the repository/repositories and accession number(s) can be found in the article/Supplementary Material.

## Ethics Statement

The studies involving human participants were reviewed and approved by the ethics committee of the Faculty of Medicine, Tokyo Medical and Dental University (M2017-208) and the ethics committee of Noguchi Memorial Institute for Medical Research, University of Ghana (FWA 00001824). Written informed consent to participate in this study was provided by the participants’ legal guardian/next of kin.

## Author Contributions

AAy, AAb, and RS conceived and designed the experiments. AAy, AK, MS, WS, SM, IP, MM, and RS performed the experiments. AAy, MS, TH, TS, SI, AAb, and RS analyzed the data. MS, KM, TH, TS, SI, AAb, and RS contributed to reagents, materials, and analysis tools. AAy, MS, AAb, and RS contributed to the writing of the manuscript. All authors contributed to the article and approved the submitted version.

## Conflict of Interest

The authors declare that the research was conducted in the absence of any commercial or financial relationships that could be construed as a potential conflict of interest.

## Footnotes

1 https://pubmlst.org/abaumannii/

2 http://halst.nhri.org.tw/PathoBacTyper/index.jsp

3 https://isfinder.biotoul.fr/
