# Supplementary material for: Prevalence and Characterization of Carbapenem-Hydrolyzing Class D β-Lactamase-Producing Acinetobacter Isolates From Ghana
Source: Front Microbiol. 2020 Nov 13;11:587398. doi: 10.3389/fmicb.2020.587398 (PMC7691484; doi:10.3389/fmicb.2020.587398)
Supplement: Supplementary file 1 [file Data_Sheet_1.docx]

# **Supplemental Tables**

**Prevalence and characterization of carbapenem-hydrolyzing class D β-lactamase-producing *Acinetobacter* isolates from Ghana**

Alafate Ayibieke^1^, Ayumi Kobayashi^1^, Masato Suzuki^2^, Wakana Sato^1^, Samiratu Mahazu^1,3^, Isaac Prah^1,4^, Miyuki Mizoguchi^5^, Kyoji Moriya^5^, Takaya Hayashi^4^, Toshihiko Suzuki^6^, Shiroh Iwanaga^3^, Anthony Ablordey^7^, Ryoichi Saito^1^*

^1^Department of Molecular Microbiology, Tokyo Medical and Dental University, Tokyo, Japan.

^2^Antimicrobial Resistance Research Center, National Institute of Infectious Diseases, Tokyo, Japan.

^3^Department of Environmental Parasitology, Tokyo Medical and Dental University, Tokyo, Japan.

^4^Department of Molecular Virology, Tokyo Medical and Dental University, Tokyo, Japan.

^5^Department of Infection Control and Prevention, The University of Tokyo Hospital, Tokyo, Japan.

^6^Department of Bacterial Pathogenesis, Tokyo Medical and Dental University, Tokyo, Japan.

^7^Bacteriology Department, Noguchi Memorial Institute for Medical Research, University of Ghana, Accra, Ghana.

*Corresponding author

Ryoichi Saito

E-mail: r-saito.mi@tmd.ac.jp

**Table S1. Quality filtered reads statistics**

| **Sample** | **Reads** | **Number of sequences** | **Sum of Length** | **Minimum Length** | **Average Length** | **Maximum Length** |
| --- | --- | --- | --- | --- | --- | --- |
| Ab-B004a-c | NBSEQ_1 | 15,292,942 | 2,232,258,257 | 31 | 146 | 146 |
|  | NBSEQ_2 | 15,292,942 | 2,232,269,560 | 15 | 146 | 146 |
|  | MinION | 266,360 | 1,818,283,492 | 1000 | 6,826.4 | 133,657 |
| Ab-D10-a-a | NBSEQ_1 | 13,807,493 | 2,015,453,169 | 24 | 146 | 146 |
|  | NBSEQ_2 | 13,807,493, | 2,015,464,053 | 15 | 146 | 146 |
|  | MinION | 251,147 | 1,848,731,729 | 1000 | 7,361.2 | 143,031 |
| Ab-C102 | NBSEQ_1 | 15,061,973 | 2,198,726,363 | 31 | 146 | 146 |
|  | NBSEQ_2 | 15,061,973 | 2,198,737,880 | 20 | 146 | 146 |
|  | MinION | 121,146 | 960,798,950 | 1000 | 7,930.9 | 146,926 |
| Ab-C63 | MiSeq_1 | 1,186,153 | 178,614,343 | 30 | 150.6 | 155 |
|  | MiSeq_2 | 1,186,153 | 178,579,576 | 30 | 150.6 | 155 |
|  | MinION | 183,129 | 1,265,401,702 | 1000 | 6,909.9 | 141,603 |

**Table S2. *De novo* assembly statistics**

| **Sample** | **Chromosome/Plasmids** | **Length (bp)** | **Circular/Linear** | **GC (%)** | **Average read depth** | **Assembler** |
| --- | --- | --- | --- | --- | --- | --- |
| Ab-B004d-c | Chromosome | 4,091,477 | Circular | 39.1 | 972.8 | unicycler |
|  | pAb-B004d-c_1 | 48,239 | Circular | 36.8 | 1238.8 | unicycler |
|  | pAb-B004d-c_2 | 8,495 | Circular | 33.4 | 14987.9 | unicycler |
|  | pAb-B004d-c_3 | 8,215 | Circular | 37.9 | 17121.5 | unicycler |
|  | pAb-B004d-c_4 | 2,697 | Circular | 36.8 | 49223.2 | unicycler |
| Ab-D10-a-a | Chromosome | 4,100,469 | Circular | 39.1 | 884.5 | flye |
|  | pAb-D10-a-a_1 | 48,239 | Circular | 36.8 | 1089.3 | unicycler |
|  | pAb-D10-a-a_2 | 8,495 | Circular | 33.4 | 13163.7 | unicycler |
|  | pAb-D10-a-a_3 | 8,215 | Circular | 37.9 | 15392.0 | unicycler |
|  | pAb-D10-a-a_4 | 6,619 | Circular | 36.2 | 298.3 | unicycler |
|  | pAb-D10-a-a_5 | 2,697 | Circular | 36.7 | 37432.6 | unicycler |
| Ab-C102 | Chromosome | 3,763,047 | Circular | 39.2 | 1113.2 | unicycler |
|  | pAb-C102_1 | 90,089 | Circular | 41.0 | 974.2 | unicycler |
|  | pAb-C102_2 | 67,097 | Circular | 38.1 | 107.5 | flye |
|  | pAb-C102_3 | 19,853 | Circular | 35.0 | 3093.3 | flye |
| Ab-C63 | Chromosome | 3,873,866 | Circular | 39.0 | 86.9 | unicycler |
|  | pAb-C63_1 | 81,353 | Circular | 41.0 | 127.5 | unicycler |
|  | pAb-C63_2 | 10,662 | Circular | 35.2 | 558.1 | unicycler |
